# Prescription Writing

**Published:** 1878-06

**Authors:** 


### BIBLIOGRAPHICAL.

---

PRESCRIPTION WRITING.—Designed for the use of medical students who have never studied Latin. By Fredrick Henry Gerrish, M.D., Professor of Materia Medica and Therapeutics in the Medical School of Maine, etc. Portland, Me.: Loring, Short & Harmon. Philadelphia: J. B. Lippincott & Co. 1878.

This littte volume is designed to give instruction to those who have not a knowledge of Latin, in the principles of prescription writing—especially as relates to the terminology of the pharmacopœia. It contains a full list of the declensions of the nouns, together with an explanation of the meaning of many Latin words and phrases used in prescription writing. It is not a book of formulæ—it only teaches *how* to write prescriptions in Latin. We do not hesitate in commending it to those for whom it is intended.

